# Health Impact Assessment of Indira Sagar Project: a paramount to studies on Water Development Projects

**DOI:** 10.1186/s12936-017-1688-0

**Published:** 2017-01-26

**Authors:** Anushrita, B. N. Nagpal, Neera Kapoor, Aruna Srivastava, Rekha Saxena, Shailendra Singh, Sanjeev Gupta, Sompal Singh, Kumar Vikram, Neena Valecha

**Affiliations:** 1 National Institute of Malaria Research (ICMR), New Delhi, India; 2 Indira Gandhi National Open University, New Delhi, India; 3 National Rural Health Mission, Bhopal, MP India

**Keywords:** HIA, Hydropower project, Dam, Irrigation, Malaria, *Anopheles culicifacies*, *Anopheles fluviatilis*, *Anopheles stephensi*

## Abstract

**Background:**

Very limited studies on Health Impact Assessment (HIA) of Water Development Projects (WDP) in relation to mosquito-borne diseases have been carried out in India. The current study focuses on using HIA as a tool for finding impact of Indira Sagar Project, Madhya Pradesh on human health in relation to mosquito borne diseases, and emphasizing its incorporation as an integral part of any WDP.

**Methods:**

Screening, scoping, assessment, recommendation, reporting, and evaluation were carried out in selected study areas. Entomological, epidemiological, socio-economic and knowledge, attitudes and practices data related to malaria transmission in three dam components: Submergence (SUB), Command (CMD) and Resettlement and Rehabilitation (RR) colonies were generated for the period of January 2013–December 2014. Statistical analysis was attempted to compare data among dam components and to identify risk factors. Component-specific mitigation measures were suggested based on observations.

**Results:**

*Anopheles culicifacies* was the dominating species in all three dam components and its man-hour density in CMD areas was higher compared to SUB and RR. The odds of finding a positive malaria case was much higher in CMD compared to SUB (OR 1.24, CI 95% 0.71–2.43) and RR (OR 5.48, CI 95% 0.73–40.63). Respondents of CMD stated more previous episodes of malaria (81.8%) compared to RR (61.4%) and SUB (55.7%). The canonical discriminant analysis concluded that distance from reservoir/Indira Sagar canal had the highest discriminating ability of malaria cases in different components followed by treatment-seeking behaviour and malaria history. The analysis identified these risk factors with 70% accuracy.

**Conclusion:**

Engineering manipulations may be carried out in CMD areas to control seepage and RR colonies should be established beyond 3 km from reservoir/Indira Sagar canal considering the flight range of *A. culicifacies*. Strengthening of surveillance with early detection and complete treatment was recommended for CMD areas. To avoid future transmission in other areas and projects HIA should be carried out at planning stage for planning better control activities.

## Background

Hydropower projects are being designed and dams constructed on an unprecedented scale. With the core aspiration of navigation, the diversion of rivers to canals for irrigation, controlling water flow during flood or drought, creating artificial lakes for fisheries and other recreational use, these highly ambitious projects aim to meet the needs of populations via the generation of hydroelectricity, increased agricultural productivity with better irrigation and supply of potable drinking water, and thereby improved socio-economic status of the beneficiaries.

To date, an estimated 45,000 large and 800,000 small dams have been constructed worldwide, covering 272 million hectares of land for irrigation, inundation and change of area of more than 500,000 sq km globally [1–3]. These changes can be both beneficial and deleterious and have both direct and/or indirect impact, depending on many factors. Being a crucial part of developing economies, dams and hydropower projects have the potential to alleviate poverty and make a positive impact on human health, although the environmental impact from construction of large dams can have repercussions on local ecology and micro-climate. At the 760th International Congress on River Basin Management, these impacts were classified under different criterion: short- versus long-term impact, impact a short or long distance from a dam, social versus non-social impact, beneficial versus harmful effects, etc.

The indirect effects become visible in terms of vector-borne diseases due to the creation of favourable breeding places and provision of optimum temperature and humidity allowing disease vectors and pathogens to flourish. Many previous studies to assess the impact on catchment areas on human health suggested that creation of favourable breeding grounds for vector mosquitoes, flies and snails inflicted once-untouched areas with many vector-borne diseases, such as malaria, lymphatic filariasis and schistosomiasis [4–10]. It was also reported that people in close proximity to dams and impoundment areas were at higher risk of vector-borne diseases compared to those living at a distance [11]. Building colossal dams not only escalates vector-borne diseases in affected areas but also alters the quality of water, resulting in the spread of water-borne diseases such as cholera, salmonellosis, amoebiasis [12]. A systematic approach to studies on Health Impact Assessment (HIA) was much needed in relation to Water Development Projects in India.

**Fig. 1:**
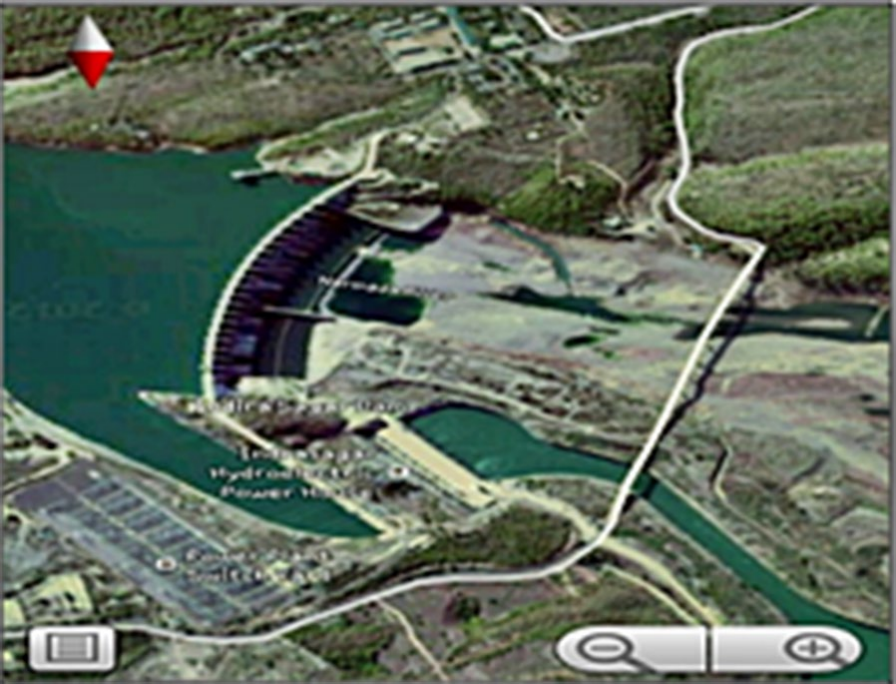
ISP Dam site, Narmada Nagar, MP

**Fig. 2:**
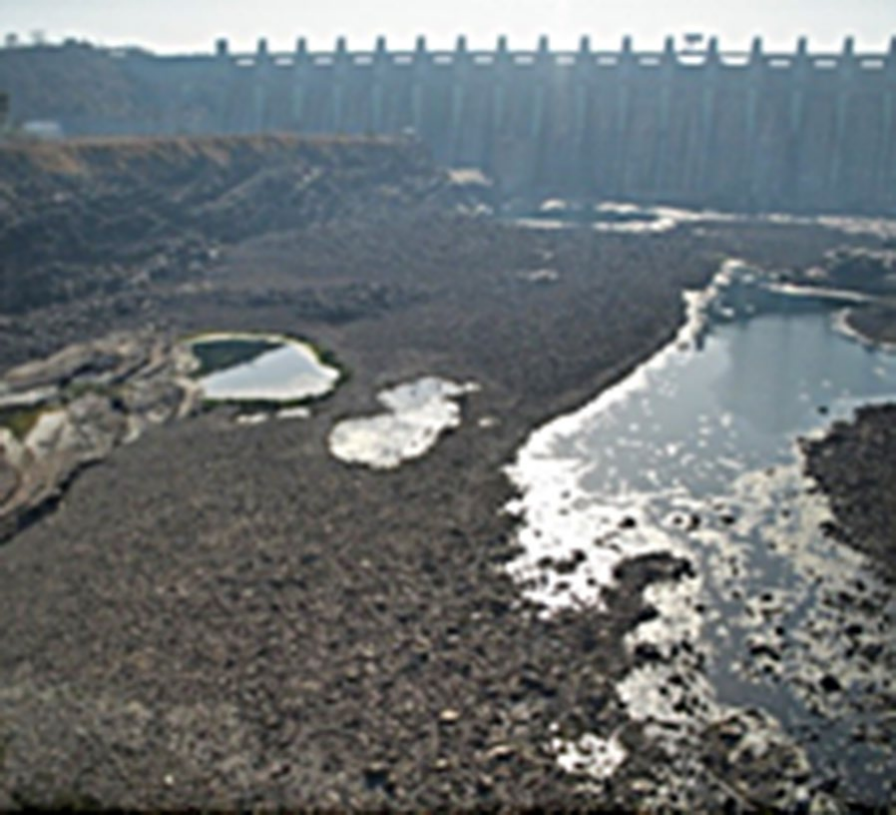
Puddles at down-stream Indira Sagar Dam, Narmada Nagar, MP

**Fig. 3:**
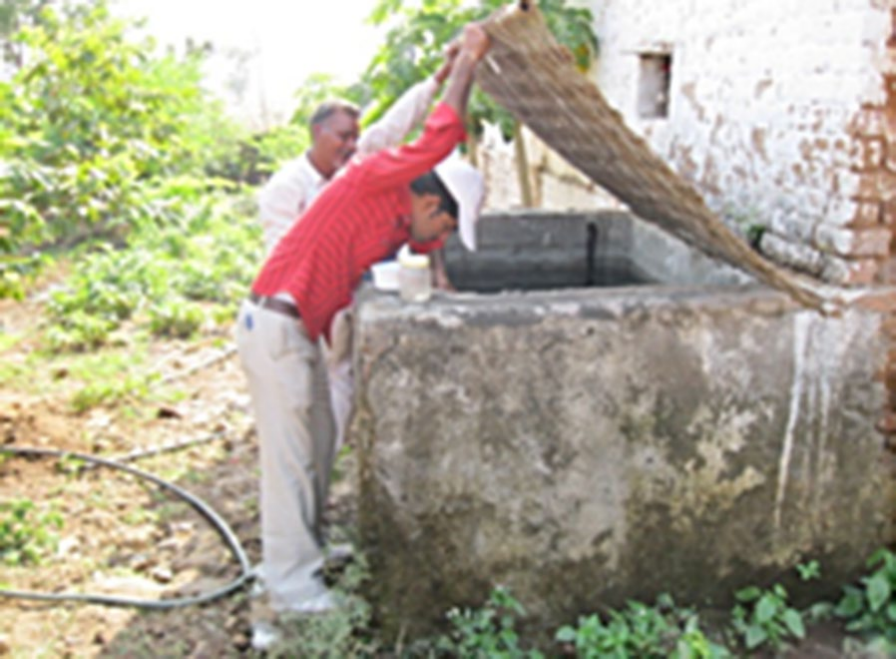
Tanks at Anjaniya RR colony, Khandwa, MP

**Fig. 4:**
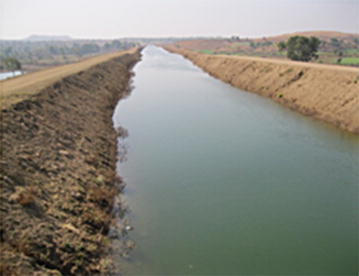
ISP main canal, Village Atarsumbha, Khargone, MP

In India, more than 4000 dams are being built for various purposes [13]. The central state of India, Madhya Pradesh (MP) comprises of 30 major dams on the River Narmada and its tributaries. Most of these dams challenge the existing fragile health infrastructure of remote areas of MP. The Indira Sagar Dam Project (ISP) was completed in 2004. It inundates a vast area and submerged many villages (as per Narmada Valley Control Authority). The construction of the 248-km main canal of ISP was designed in phases and construction work is ongoing. Consequently, ecological changes have occurred on a vast scale, providing a new avenue for vector dynamics (Figs. 1, 2, 3, 4).

According to the World Health Organization (WHO) HIA is a relatively new tool with no single internationally agreed methodology, but a recommended framework that can be carried out for a particular area by those responsible at various levels of governance (intergovernmental, governmental or regional/municipal policies), strategies, programmes or projects. HIA involves a six-step process which starts with screening, scoping, assessment, recommendations, reporting, and evaluation.

Despite the complexity of a multi-dimensional approach, HIA has an established functional framework in many developed countries, and which is now gaining momentum in developing countries [14–16]. Dedicated, uniform practice guidelines remain obscure at global level [17]. HIA has grown in the past two decades and offers a unique opportunity for solving challenges associated with any proposed project or policy. Countries such as the UK are reported to practice it as a stand-alone process. In the high human development index (HDI) countries it has been used extensively, emphasizing the inter-dependencies of various types of HIA. Although well established in high HDI countries, countries with medium and low HDI have limited applicability of HIA to large development projects [18]. HIA, as a stand-alone practice in the Indian context has been very limited. Although HIA of Water Development Projects (WDPs) has been made mandatory by the World Commission on Dams [19], it has been under the aegis of environment impact assessment (EIA) for some time. The present study was carried out to ensure the systemic practice of HIA while evaluating the burden of vector-borne diseases in relation to WDPs.

Based on a review of the literature, the current study focuses on identifying the impact of ISP on malaria transmission, along with its major risk factors, and emphasizing incorporation of HIA as an essential part of all WDPs.

## Methods

### Screening

**Fig. 5:**
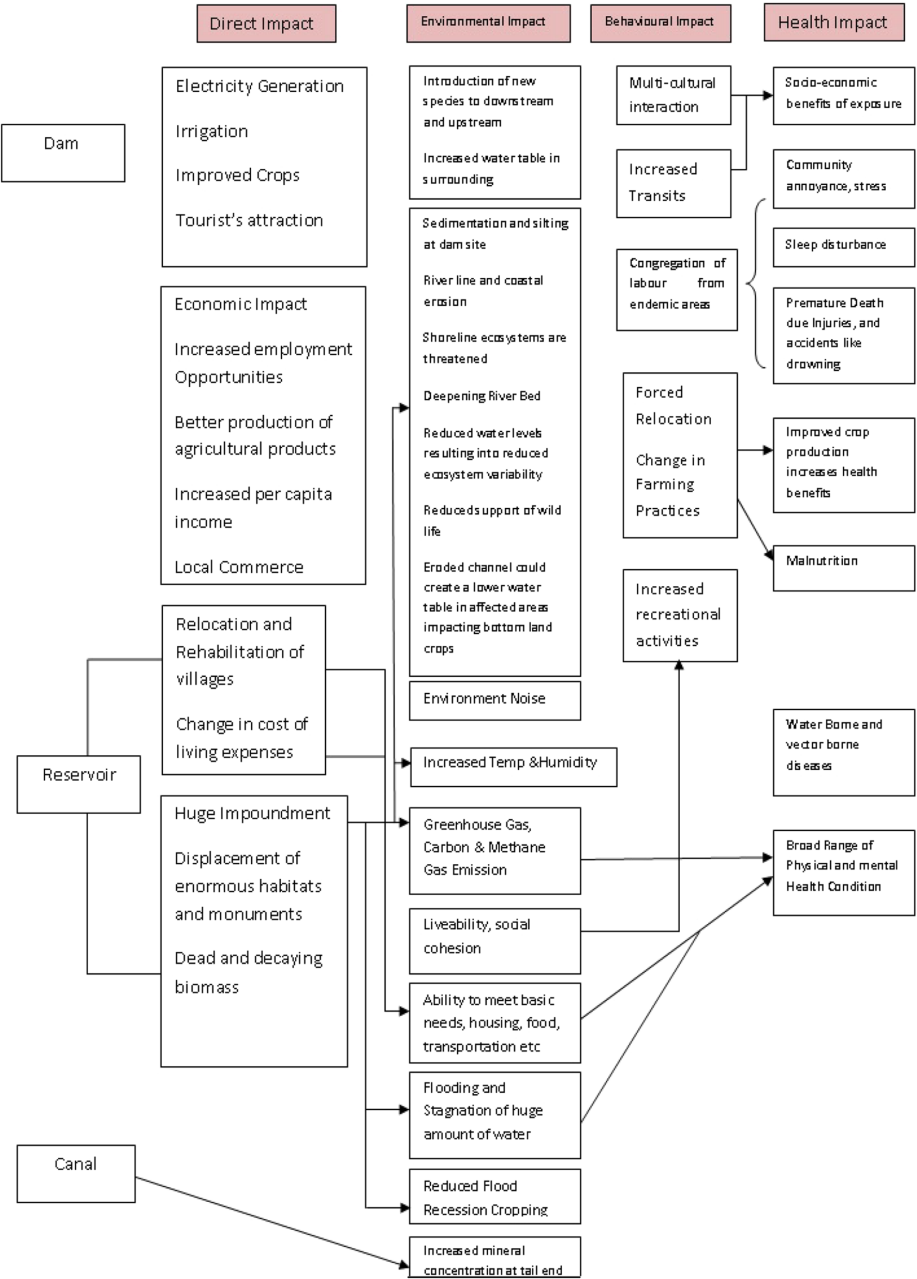
HIA Model for various aspects of impact incurred upon human life due to a hydro power plant installation in the vicinity

A rigorous review of the literature was carried out and a framework for HIA of WDPs was prepared (Fig. 5). ISP was identified with three components: SUB, CMD and RR colonies. The villages affected by ISP were classified into SUB, while villages affected by the Indira Sagar Canal were classified into CMD area. Villages under complete Submergence were shifted and resettlement was done near the Submergence areas. These were classified as RR colonies. An increase in prevalence of vector-borne diseases: schistosomiasis, encephalitis, malaria, haemorrhagic fever, gastroenteritis, intestinal parasites, and filariasis (including onchocerciasis and bancroftosis), has been reported in other geographical regions in association with WDPs [17, 20]. With available data (published and unpublished) on ISP from the state health department, it was inferred that malaria was prevalent only in the study areas.

### Scoping

**Fig. 6:**
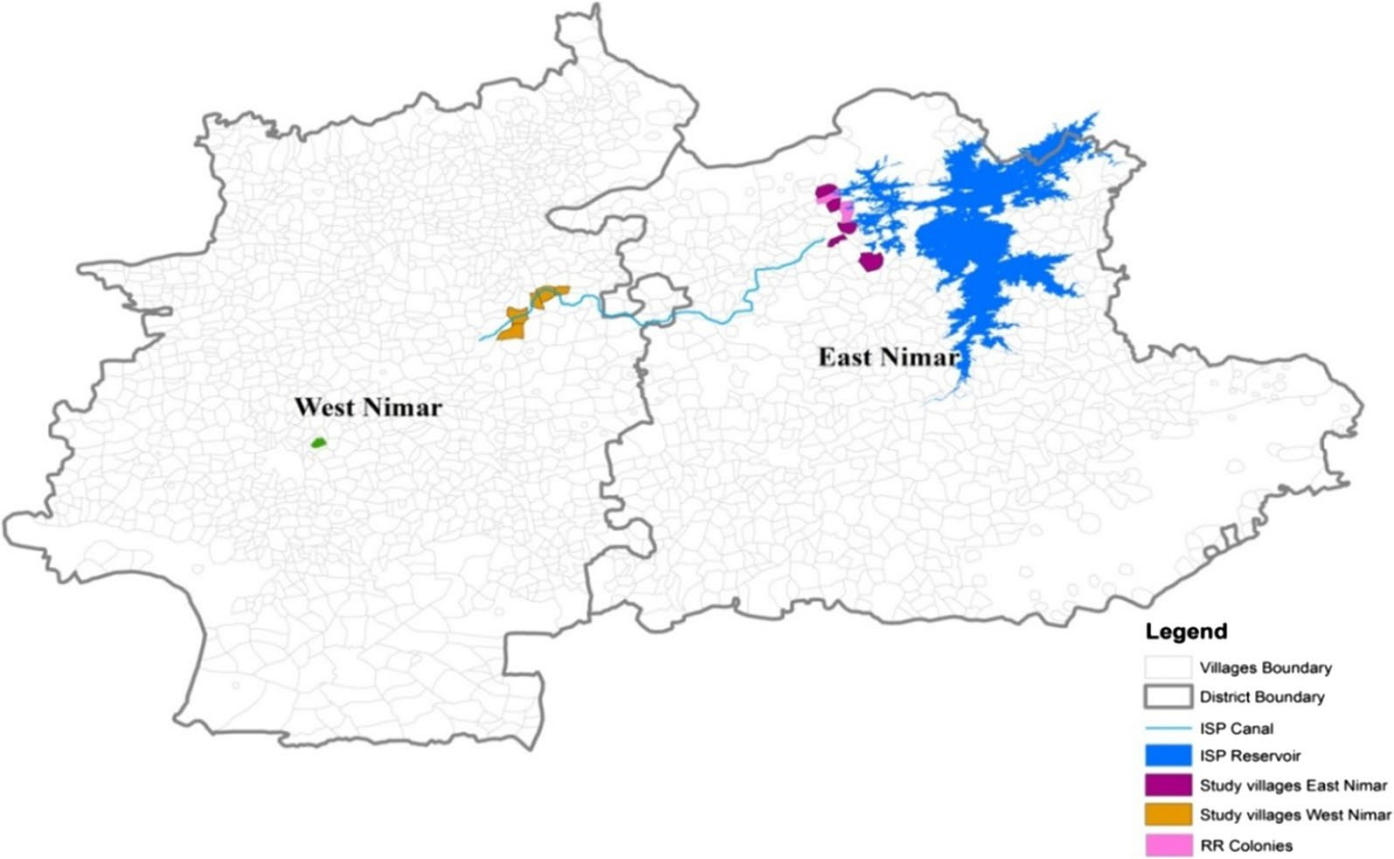
GIS map for study sites in East Nimar and West Nimar districts of MP, India

Blueprints of areas affected by ISP were collected from various engineering departments of Khandwa, Khargone and Indore to work out the areas of study. Six villages were selected from SUB, five from CMD, and two RR colonies of East and West Nimar Districts of MP (previously known as Khandwa and Khargone). The six villages selected from SUB areas were Reechi (N 22°14′642″ E 076°25′040″), Bedhani (N 22°14′162″ E 076°25′678″), Piplani (N 22°13′424″ E 076°26′305″), Chiktikhal (N 22°10′687″ E 076°27′339″), Chandel (N 22°11′840″ E 076°27′300″), and Jamkota (N 22°08′311″ E 076°29′590″); from CMD, the selected villages were Guradiya (N 22°04′964″ E 075°59′316″), Piprikheda (N 22°02′996″ E 075°58′026″), Atarsumbha (N 22°03′839″ E 75°59’146″), Mokhangaon (N 22°05′994″ E 076°00′665″), Birali (N 22°05′587″ E 076°01′735″); from RR colonies: Bedhani (N 22°14′161″ E 76°25′123″) and Anjaniya (N 22°12′018″ E 76°26′515″) (Fig. 6). The selection was based on the flight range of vector mosquitoes falling under 3 km from SUB and CMD areas.

### Assessment

Data on epidemiological, entomological, socio-economic, and attitudes and practices (KAP) related to malaria transmission in three dam components: SUB, CMD and RR colonies, were generated for the period of January 2013–December 2014.

### Entomological data collection

**Fig. 7:**
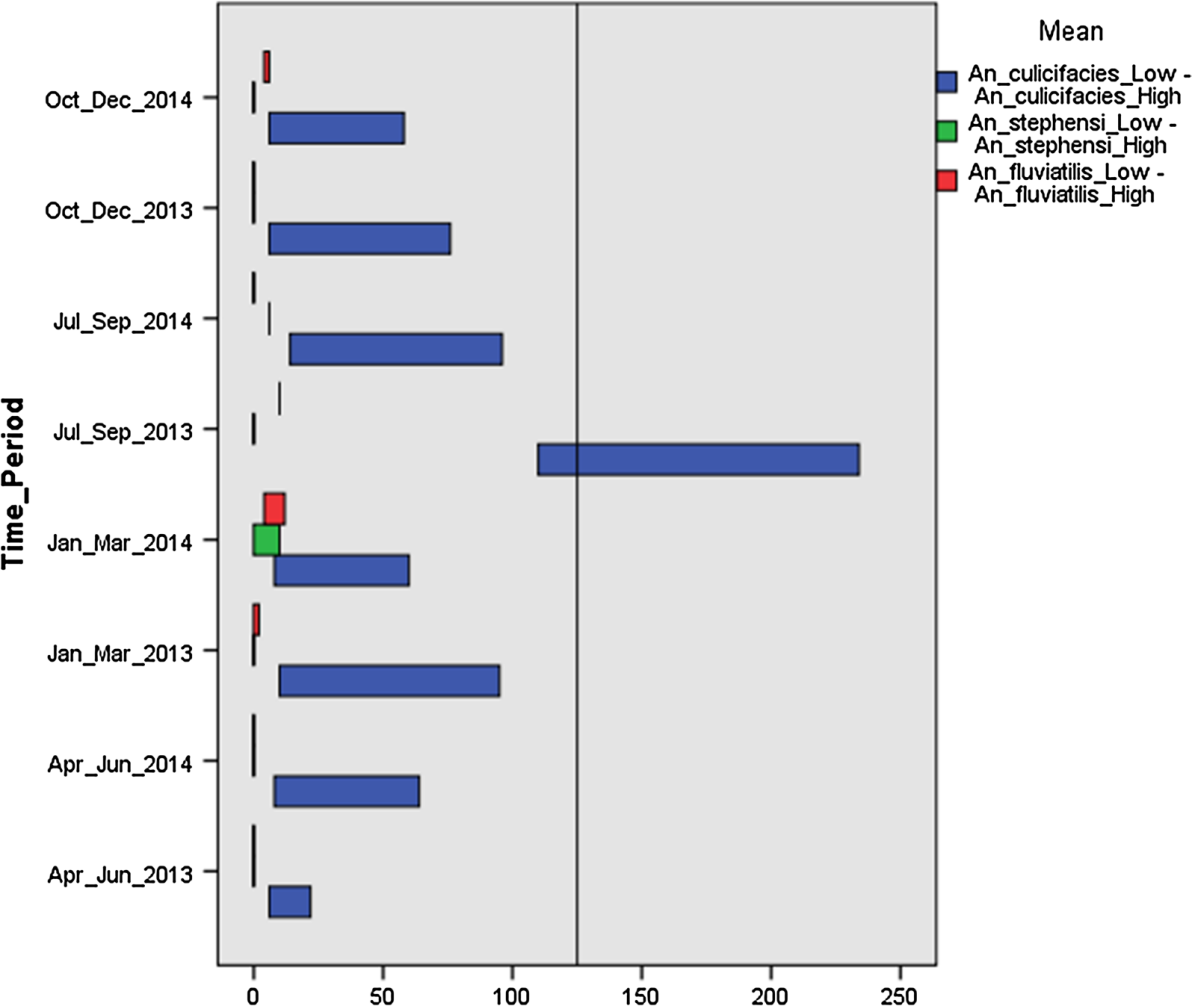
Range of minimum and maximum Man Hour Density (MHD) of major malaria vectors was calculated in selected villages for the period of 2013–2014

For logistical reasons, bimonthly collection of entomological data (adult mosquitoes and larvae) from all the villages was done. To cover each entire village, four fixed stations and three randomly selected houses and cattlesheds were selected during each survey. Adult collection was done early morning (06.00–07.00) with permission of house-members. Hand-catch collection in their houses was carried out with the help of a torch and a suction tube. Mosquitoes were caught for an hour as per WHO guidelines. The collected mosquitoes were brought to the insectary at Narmada Nagar, Khandwa District for further identification and the range of minimum and maximum MHD was calculated. MHD of mosquitoes was calculated in terms of total number of mosquitoes collected by each person per hour. Data collected on MHD were pooled quarterly and comparative lows and highs are presented in Fig. 7 for 2013 and 2014. The vector species were identified using standard key by Nagpal and Sharma [22]. Data on larvae collection and emergence of various vector mosquito species are published separately [23].

### Collection of blood slides and identification of malaria parasites

Blood slides were prepared by finger-prick during door-to-door surveillance (patients with fever) and were brought to Narmada Nagar for identification of parasite species. Thick and thin blood smears were stained by standard JSB Stain-1 and 2 and were identified under microscope with oil immersion at 100X magnification. Rapid diagnostic tests were done for selected cases and data were corroborated by slide examination. Positive cases were immediately informed for further treatment and follow-up. The state health authorities were well equipped with treatment facilities.

### Socio-economic and KAP data collection

For this cross-sectional study, sample size for KAP study was determined using the formula provided by Charan and Biswas for 95% CI [24].

### Data collection

In order to collect socio-economic and KAP data related to malaria in selected components of ISP, a structured questionnaire was prepared and translated into Hindi. The questionnaire comprised: (a) age, sex, education, occupation, and annual income; b) history of malaria; (c) knowledge of symptoms of malaria fever; (d) knowledge of vector breeding sites; (e) treatment-seeking behaviour; and, (f) use of self-protection measures. A total of 209 participants were interviewed from approximately 600 households. Unwillingness to respond was attributed to many factors, such as unawareness of importance of studies, indecisiveness, cultural practices and social obligations.

### Data analysis

Statistical analysis was attempted to compare data among dam components. Multivariate canonical discriminant analysis was separately attempted in SPSS (version 20) to identify risk factors. Multiple variables were tested for their relation to malaria prevalence in three components and a canonical discriminant model was used in SPSS incorporating the following variables: (a) location of malaria positive case in different dam components from reservoir/Indira Sagar Canal; (b) occupation; (c) house type; (d) level of education; (e) treatment-seeking behaviour; and, (f) history of malaria fever.

### Recommendations and reporting

Based on observations specific to engineering, epidemiology and entomology, component-specific recommendations based on principles of integrated vector management [21], such as source reduction, community mobilization, collaboration with other stake holders, and capacity building, were made for the study areas of ISP.

### Evaluation

Evaluation was done by various stakeholders: Narmada Valley Development Authority (NVDA), National Hydro Development Corporation (NHDC) and the Independent Committee of Ministry of Environment and Forest, India.

## Results

Studies on MHD revealed presence of *Anopheles culicifacies*, *Anopheles stephensi*, *Anopheles fluviatilis*, *Anopheles subpictus*, and *Anopheles annularis* in the study area. The average MHD of all the mosquitoes was observed to be lower in 2014 compared to 2013. A gradual increase in MHD was observed during the monsoon followed by a decline in post-monsoon (October–February) to pre-monsoon (March–May). MHD in three components varied in all quarters, and villages of CMD area had more vector density compared to SUB and RR colonies. *A. culicifacies* was found to be the dominating species in all three dam components and its MHD in CMD areas was higher compared to SUB and RR.

During active fever surveillance, a total of 3308 slides were prepared from all the study villages during 2013–2014 and a total of 53 malaria-positive cases were found. Maximum positive cases were found in villages of CMD areas (17 *Plasmodium falciparum* and ten *Plasmodium vivax* cases), followed by SUB (seven *P. falciparum* and 18 *P. vivax* cases), and RR colonies (one *P. vivax* case). Odds of finding a positive malaria case were higher in CMD compared to SUB (OR 1.24, CI 95% 0.71–2.43) and RR (OR 5.48, CI 95% 0.73–40.63).

Analysis of socio-economic and KAP data indicated that education in all three components was limited mainly to primary education. Only 0.89% respondents from SUB, 7% from CMD and 9% from RR colonies completed their graduation. There was a significant difference in the annual income of all three components. Difference in annual income in RR and SUB was found statistically significant from CMD income (Pearson’s χ^2^ = 20.45, p = 0.025).

Housing pattern varied significantly in these components (Pearson’s χ^2^ = 9.79, p = 0.04); 58.4% houses in SUB were *kutchha* (uncemented) while 18.6% were cemented with roofing with asbestos; 72.2% houses in CMD were *kutchha* and 14.8% were cemented while in RR colonies 38.5% houses were cemented and 17.9% were *kutchha*.

There was a significant difference in knowledge of causal organism for spreading malaria (Pearson’s χ^2^ = 28.20, p = 0.002). All respondents from CMD were sure about their knowledge of mosquitoes being the causal organism for spreading malaria. Most of the people assumed that malaria mosquitoes breed in mud and soil. Another significant difference was observed in personal protective measures as only 30.8% respondents from RR used bed nets as a protective measure compared to 13% from CMD and 8% from SUB (Pearson’s χ^2^ = 27.77, p = 0.00).

**Fig. 8:**
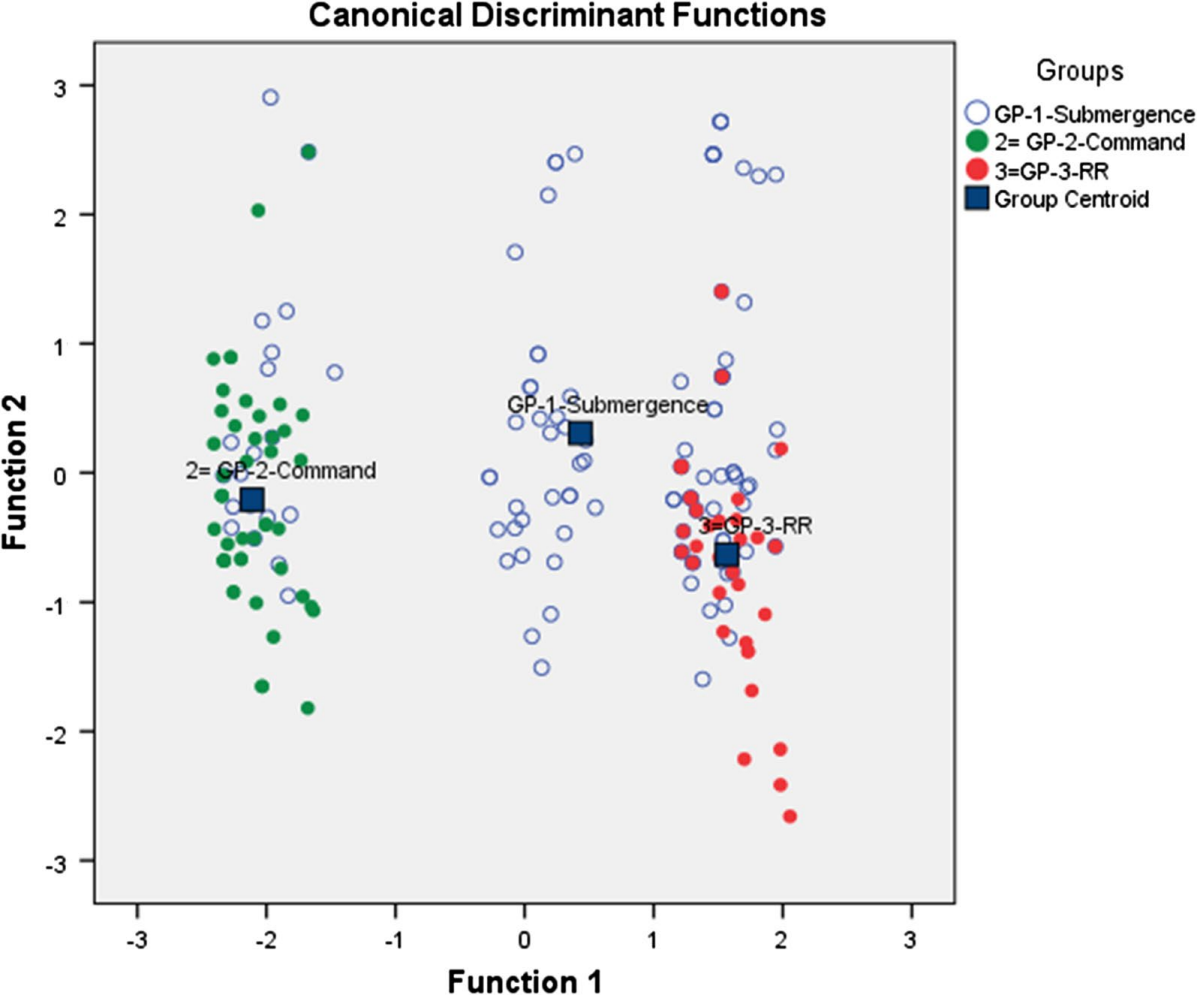
Discriminant analysis of selected villages based on specific set of variables—cases from all components are dispersed in different colors around the group centroid calculated by canonical discriminant functions

Respondents from CMD preferred to go to Government hospitals for treatment while villagers of SUB and RR colonies preferred private clinics (Pearson’s χ^2^ = 89.24, p = 0.00). There was a significant difference in previous episodes of malaria in respondents of all the three groups (Pearson’s χ^2^ = 75.37, p = 0.00). Respondents in CMD had more previous episodes of malaria (81.8%) compared to RR (61.4%) and SUB (55.7%). The canonical discriminant model concluded that distance from reservoir/Indira Sagar Canal had the greatest discriminating ability of malaria cases in different components followed by treatment-seeking behaviour and malaria history. The model identified these risk factors with 70% accuracy (Fig. 8; Table 1).

**Table 1:** Classification results of canonical discriminant analysis

Classification results^a^				
Groups	Predicted group membership			Total
	GP-1-Submergence	2 = GP-2-Command	3 = GP-3-RR	
Original				
Count				
GP-1-Submergence	54	20	36	110
2 = GP-2-Command	0	50	0	50
3 = GP-3-RR	3	0	34	37
%				
GP-1-Submergence	49.1	18.2	32.7	100.0
2 = GP-2-Command	.0	100.0	.0	100.0
3 = GP-3-RR	8.1	.0	91.9	100.0

^a^70.1% of original grouped cases correctly classified

## Discussion

Comparing previous health data, it was found that there were no malaria cases before construction of dams and canals in these areas. It was apparent from the study that ISP dam components make an important contribution to malaria transmission in MP. Various aspects of a WDP were explored while conducting this HIA and based on observations, component-specific mitigation measures were suggested in detail to NVDA and other stakeholders such as the state health department and NHDC.

MHD of malaria vector mosquitoes: *A. culicifacies, A. stephensi* and *A. fluviatilis,* varied considerably among different components. Formation of new irrigation channels or uplift structures to provide water to villages from the main canal was another challenge in the area. The water channels with percolating base allowed growth of lichens and other vegetation, which provided further anchorage for mosquito larvae. *A. culicifacies* was the dominating species in all three dam components and its MHD in CMD areas was higher due to these factors compared to SUB and RR. Another reason for higher MHD in CMD areas as observed in these areas was due to the proximity of houses to the canal network. Seepage and stagnation of water in these channels provided ample breeding grounds for malaria vectors*. A. fluviatilis* was breeding due to formation of small, flowing streams from seepage in CMD/ SUB areas. *A. stephensi* was breeding in RR colonies due to gradual formation of cemented tanks. For villages affected in SUB, the level of reservoir changes and fluctuates during monsoon and post-monsoon: the reservoir is full during monsoon, with water reaching very close to the villages at the periphery. This water recedes post-monsoon when the villages were observed to have reduced mosquito density. To rehabilitate the SUB villages, RR colonies have been built within 2–3 km of ISP. Although these RR colonies provide better housing compared to both SUB and CMD areas, yet their closer proximity to the reservoir was found to be a major risk factor during monsoon when water levels increase, as evident by the result of canonical discriminant analysis. Considering the flight range of *A. culicifacies*, it has been recommended to establish RR colonies beyond 3 km of reservoir/ISP canal.

CMD areas reported more malaria cases compared to SUB and RR areas during active fever surveillance. This was confirmed while recording previous episodes of malaria cases during KAP data collection in CMD areas. The higher number of malaria cases in CMD areas can be attributed to the frequent host-vector interaction due to proximity of *A. culicifacies* breeding grounds compared to SUB and RR colonies. This study is congruent with findings of Kibret et al. [25] with its evidence of increased transmission of malaria in irrigated villages compared to non-irrigated villages of Ethiopia. Similar to present studies, Vas dev and Sharma reported an increase in breeding grounds of malaria vectors in rural India [26]. The proximal breeding grounds, frequent influx of labour for construction work from endemic areas into CMD areas of ISP main canal are the possible reasons for an intermittent rise in malaria-positive cases, along with annual transmission. Strengthening of surveillance with early detection and complete treatment was recommended for CMD areas. In a similar but more exhaustive study carried out in Nam Theun 2 hydroelectric project (NT2) in the Lao People’s Democratic Republic, it was observed that malnutrition and infections with intestinal nematodes were of major concerns contrary to vector-borne diseases, such as malaria. Better housing in resettled areas, which were provided with screening from vectors, were found to be an important reason for this [27].

Differences in all three dam components provide the evidence that targeted interventions are required for reduction in component-specific disease morbidity. The importance of inter-sectoral collaboration for maximizing the benefits of the dams while minimizing the associated risks is crucial. To maximize the health benefit of every large dam, a detailed baseline survey should be a part of its assessment and a comprehensive action plan should be implemented to limit adverse impact [28]. Scott-Samuel and World Bank advocate the implicit role of comprehensive impact assessment to ensure positive outcomes of development projects [29, 30]. Although the Government of India, National Malaria Control Programme is functional, a strong focus of state government in areas of ISP dam components would play a key role in control. Out of 30 major dam projects in Narmada Basin, some are under construction and the canal areas take most time to be completed. HIA of such WDPs during their early stages will help to foresee changes in the affected areas and ready health systems to fight against any uncertainty and take timely actions before it is too late.

## Conclusion

Regular and component-specific monitoring and intervention in dam impoundments and their irrigation channels are a mandate to check on any sudden upsurge of malaria and other vector-borne diseases. Keeping in mind the flight range of the rural vector *A. culicifacies*, RR colonies constructed to provide better accommodation should be beyond 3 km from any dam component. With increasing complexities due to an upsurge in travelling and migration habits, malaria can extend to places where it had been expected to be eliminated. With growing demand for food and electricity, construction of dams and impoundments are speculated to increase in coming years. In the absence of specific control strategies for dam components they could be more of a bane than a boon to their beneficiaries. A prudent decision-making tool such as HIA with the consent of all stakeholders and a timely strategy will result in a sustainable action plan for all affected areas of an impoundment.
